# Use of pilot plant scale continuous fryer to simulate industrial production of potato chips: thermal properties of palm olein blends under continuous frying conditions

**DOI:** 10.1002/fsn3.76

**Published:** 2013-11-27

**Authors:** Azmil Haizam Ahmad Tarmizi, Razali Ismail

**Affiliations:** Malaysian Palm Oil Board6 Persiaran Institusi, Bandar Baru Bangi, 43000, Kajang, Selangor, Malaysia

**Keywords:** Binary blends, continuous frying conditions, potato chips, qualitative parameters, stability

## Abstract

Binary blends of palm olein (PO) with sunflower oil (SFO), canola oil (CNO), and cottonseed oil (CSO) were formulated to assess their stability under continuous frying conditions. The results were then compared with those obtained in PO. The oil blends studied were: (1) 60:40 for PO + SFO; (2) 70:30 for PO + CNO; and (3) 50:50 for PO + CSO. The PO and its blends were used to fry potato chips at 180°C for a total of 56 h of operation. The evolution of analytical parameters such as tocols, induction period, color, *p*-anisidine value, free fatty acid, smoke point, polar compounds, and polymer compounds were evaluated over the frying time. Blending PO with unsaturated oils was generally proved to keep most qualitative parameters comparable to those demonstrated in PO. Indeed, none of the oils surpassed the legislative limits for used frying. Overall, it was noted that oil containing PO and SFO showed higher resistance toward oxidative and hydrolytic behaviors as compared to the other oil blends.

## Introduction

Frying is an ancient and well-established process in food preparation. This is evidenced by a great increase in fried food consumption in the recent years, of which more than 20 million tonnes of world annual oil production is extensively utilized for frying (Gertz 2004). The process is essentially a dehydration of food that involves rapid heat and mass transfer in food immersed in hot oil at temperatures greater than the boiling point of water (Farkas et al. 1996).

Despite significant amount of oil being absorbed by the food, frying is extensively employed in domestic as well as industrial practice, due to its ability to create unique sensory properties—for example, texture, flavor, and taste—which make the food more palatable and desirable (Ahmad and Ismail 2007). In addition, its operational simplicity, convenience, and economic viability have resulted in extensive sales of a large variety of fried products. It is worth noting that frying oil serves up a high source of energy of up to 9 kcal g^−1^, more than double than the energy derived from carbohydrates and proteins. Frying oil is also a source of lipid soluble vitamins (A, D, E, and K), and provides essential fatty acids such as oleic, linoleic, and linolenic acids, which are essential for human metabolisms.

Stability is always associated with the ability of oil to withstand high frying temperatures. Due to that reason, the degree of saturation becomes a major concern for choosing the right oil for frying (Berger 2005). In general, oil containing higher level of saturated fatty acids is likely to be more stable for frying, for example, palm oil. Oils enriched with polyunsaturated fatty acids or so-called soft oils such as sunflower, soybean, and safflower oils are less stable in comparison to oils having higher level of monounsaturated and saturated fatty acids. Cottonseed oil (CSO) seems to be more stable than other soft oils as a result of relatively higher saturated fatty acids.

Due to this reason, a lot of research were undertaken to improve the stability of oil. This includes alteration of fatty acids composition by blending, hydrogenation, or plant breeding. Hydrogenation is a process of flushing hydrogen into the oil at elevated temperature and pressure in the presence of suitable catalysts such as nickel. Nevertheless, considerable amount of *trans* fatty acids present in the hydrogenated oil is nutritionally undesirable (Kochhar 2001). Modification in fatty acids profile through breeding is also explored to improve the stability of soft oils, that is, high oleic sunflower oil (SFO), high oleic canola oil (CNO), and high oleic soybean oil (Rossell 2001). Performances of high oleic oils for frying have been reported in literatures; however, the advantages of such oils is somehow denied by limited supply and resulted in higher trading price. Therefore, it is believed that blending is the most favored among others as the process is direct and cheaper, without having to concern about formation of *trans* fatty acid when the oil undergoes for hydrogenation (Naghshineh and Mirhosseini 2010).

Many publications have reported the performance of vegetable oils during frying. Nevertheless, almost all of the studies were performed under batch or intermittent frying conditions, and surprisingly published papers that simulate industrial (continuous) frying, by far, does not even exceed 10 articles (Sebedio et al. 1996; du Plessis and Meredith 1999; Kristott 2000; Ahmad Tarmizi & Ismail, 2008; Ismail 2005). This is probably due to higher cost associated with continuous frying trials and/or trade secret of the food industry to protect their products. It is also worth mentioning that the two frying protocols have totally different process operations in terms of degree of oil deterioration and frying performance.

Palm oil, particularly its liquid fraction palm olein, is extensively used in frying sectors. The oil is normally regarded as heavy duty oil because of its technoeconomic advantages over other vegetable oils; this can be explained from the basis of its stronger heat resistance and competitive trading price (Nallusamy 2006). Despite all this, the prospect of palm olein seems not convincing enough to some food processors and consumers not only because of perceptions on issues associated with saturated oils but also related to the preference of their local oils. Realizing the limitation of unsaturated oils in the perspective of stability, one of the routes to penetrate the use of palm olein, particularly for industrial frying, is through blending. Frying stability of palm olein blends is widely discussed (de Marco et al. 2007; Khan et al. 2008; Farhoosh et al. 2009; Abdulkarim et al. 2010; Naghshineh and Mirhosseini 2010; Al-Khusaibi et al. 2012); nevertheless, this is not in the case of continuous frying. Therefore, the study attempts to evaluate the performance of blends of palm olein with other vegetable oils such as SFO, CNO, and CSO in the form of binary mixtures under continuous frying conditions. The results gained would benefit the snack food processors who are looking for stable oils, particularly for use in industrial frying, without the need to fully replace their preference oils with palm olein.

## Material and Methods

### Raw materials

Refined, bleached, and deodorized (RBD) palm olein (PO) was processed by Golden Jomalina Food Industries Sdn. Bhd. (Banting, Malaysia) while RBD SFO, CNO, and CSO were purchased from MOI Foods Sdn. Bhd. (Pulau Indah, Malaysia). Potatoes of Atlantic variety were imported from Australia and kept in a refrigerator at 4°C prior to use.

### Preparation of palm olein blends

The oil blends were prepared by mixing: (1) 60% PO 40% SFO, (2) 70% PO and 30% CNO, and (3) 50% PO, and 50% CSO. The oil ratios in the case of (1) and (3) were selected to obtain an equivalent proportion between saturated, monounsaturated, and polyunsaturated fatty acids whereby incorporation CNO with 70% PO gave blended oil containing 2% linolenic acid; this process will be detailed in the Results and Discussion section.

### Frying protocols and oil sampling

Frying trials were carried out using a 200-L capacity pilot plant continuous fryer (Heat and Control Inc., Brisbane, Australia). Oil was transferred from the fresh oil tank into the fryer prior to gradual heating at 180°C using heat exchangers. Potatoes were washed, peeled, and cut into slices of 1.6 mm thickness to ensure the uniformity of heat transfer during frying. Potato slices were reconditioned using a rotary perforated drum to remove adhered starch from the surface and dried under air knife to remove excess water prior to frying for 2 min.

The production rate of potato chips was set 50 kg h^−1^ in such a way that the oil turnover time—which is the theoretical time required to fully utilize oil inside the fryer—is fixed at 10 h. Fresh oil was continuously compensated at 20 kg h^−1^ throughout the course of frying. Potato chips were collected and packed using metallized film (San Miguel Yamamura Packaging and Printing Sdn. Bhd., Shah Alam, Malaysia). For each frying trial, potato chips were processed for 8 h each day for 7 days, giving a total of 56 h of operation.

Oil was sampled from the fryer in two 500-mL dark amber bottles at predetermined time intervals, cooled, flushed with nitrogen, and stored at −20°C for subsequent physico-chemical analyses. At the end of frying operation, the oil was allowed to cool to 60°C, filtered, pumped into the used oil tank, and kept overnight under nitrogen blanketing prior to use on the next day of operation.

### Fatty acid composition

Fatty acid composition (FAC) was quantified using a Hewlett-Packard 6890 Series gas chromatography (GC) system (J & W Scientific, Folsom, CA). The fatty acid methyl ester (FAME) was prepared in accordance with AOCS Official Method Ce 1i-07 (Firestone 2009).

The GC was equipped with a flame ionization detector (FID), electronic integrator, and data processor. The flow rate of helium (carrier gas) was positioned at 0.8 mL min^−1^ with a pre-column split ratio of 1:100. A fused silica capillary column (DB-23, 60 m × 0.25 mm, i.d. 0.25 *μ*m film thickness) was fitted to the GC. The FID and injector temperature were set at 240°C. The column was initially conditioned at ambient temperature for about 1 h before being programmed to 220°C and maintained overnight until a stable base line was obtained. The temperature was then set at 180°C and remained isothermal. The fatty acid methyl esters (FAMEs) were quantified by comparing the retention times and the peak areas with that of RM-6 methyl ester standard (Supelco, Dorset, U.K.). The FAC was expressed as a percentage of each fatty acid in terms of mass fractions.

### Iodine value

Iodine value (IV) was analyzed based on the Wijs method described in the AOCS Official Method Ja 14-91 (Firestone 2009).

### Tocols

The content of tocols—tocopherols and/or tocotrienols—was analyzed in a high-performance liquid chromatography (HPLC) system (Gilson Inc., Middleton, WI), equipped with a fluorescence detector and an autosampler (Perkin Elmer, Waltham, MA), as described in AOCS Official Method Ce 8-89 (Firestone 2009).

### Induction period

Induction period was measured using a Metrohm 679 Rancimat (Metrohm AG, Herisau, Switzerland) following the AOCS Official Method Cd 12b-92 (Firestone 2009).

### Color

The color of oil samples were measured using Lovibond Tintometer Model F (The Tintometer Ltd., Salisbury, England) apparatus, in accordance to AOCS Official Method Cc 5a-40 (Firestone 2009).

### Free fatty acid

Free fatty acid (FFA) was assessed by a titration method defined in the AOCS Official Method Ca 5a-40 (Firestone 2009). The FFA was expressed as the percentage of palmitic acid.

### Smoke point

Smoke point was determined according to the Cleveland open cup method described in the AOCS Official Method Cc 9a-48 (Firestone 2009).

### *p*-Anisidine value

The *p*-anisidine value (AnV) was analyzed following the AOCS Official Method Cd 18-90 (Firestone 2009).

### Polar compounds

Polar compounds were determined gravimetrically by silica column chromatography following the IUPAC 2.507 (Dieffenbacher and Pocillinton 1992). The polar compounds were determined by deducting the mass of the sample dissolved in PE-DE mixture with the nonpolar compounds.

### Polymer compounds

Polymer compounds were identified according to the method described by Peled et al. (1975). The oil sample was initially reacted with 1% sulfuric acid in methanolic solution (Systerm, Shah Alam, Malaysia) prior to refluxing for about 2½ h. The methanolic layer was then separated from the semisolid residue after 2½ h of cooling. This insoluble viscous residue was rinsed using methanol (System, Shah Alam, Malaysia) and subsequently dissolved with chloroform (LAB-SCAN, Dublin, Ireland) before being transferred into a conical flask. Chloroform was removed using a rotary evaporator (Büchi Labortechnik AG, Flawil, Switzerland) and the remaining residue was dried to constant weight at 105°C using a convection oven (Memmert, Schwabach, Germany).

### Data evaluation

Each type of oil was evaluated in duplicate frying sessions while the oil was sampled from each session in triplicate. One-way analysis of variance (ANOVA) was carried out using SPSS 10.0 (IBM Corporation, Armonk, NY) for Windows version to compare data obtained for different frying times and oils. The differences were considered significant when *P *<* *0.05 at a confident level of 95%. Arrangement of data for statistical analysis was performed by using Microsoft Office Excel 2007.

## Results and Discussion

### Initial fatty acids composition

Fatty acid composition (FAC) is one of the direct routes to predict the stability of oils. Table 1 shows the FAC of parent oils—that is, PO, SFO, CNO, and CSO—and binary blends of PO with the three other oils. PO contains a balance proportion of saturated and unsaturated fatty acids, and this imparts to the stability of oil. With regard to soft oils, the saturation level of SFO and CNO was no higher than 10% while CSO demonstrated a slight increase in the value (27.18%). Blending PO with soft oils moderates the level of saturated fatty acids (SFA) and unsaturated fatty acids, for example, monounsaturated fatty acids (MUFA) and polyunsaturated fatty acids (PUFA) which help to improve their frying resistance against high frying temperatures.

**Table 1:** Fatty acid composition of parent and blended oils.

Fatty acids (%)	PO	SFO	CNO	CSO	PO + SFO	PO + CNO	PO + CSO
C12:0	0.26 ± 0.04a	0b	0bA	0.19 ± 0.03a	0.23 ± 0.03a	0.27 ± 0.06a	0.23 ± 0.05a
C14:0	1.18 ± 0.03a	0b	0.09 ± 0.02c	0.79 ± 0.08d	0.56 ± 0.05e	0.93 ± 0.12f	1.04 ± 0.11af
C16:0	40.82 ± 0.32a	5.84 ± 0.12b	5.08 ± 0.15c	23.44 ± 0.45d	26.83 ± 0.55e	29.65 ± 0.62f	32.08 ± 0.54g
C16:1	0.28 ± 0.04ad	0.13 ± 0.04b	0.27 ± 0.03a	0.50 ± 0.09c	0.19 ± 0.05bde	0.18 ± 0.04be	0.25 ± 0.04ade
C18:0	3.80 ± 0.13af	3.28 ± 0.11b	2.04 ± 0.13c	2.48 ± 0.10d	4.22 ± 0.21e	3.12 ± 0.34b	3.34 ± 0.58bf
C18:1	41.61 ± 0.45a	38.75 ± 0.92b	60.17 ± 0.79c	16.59 ± 0.47d	35.86 ± 0.80e	47.44 ± 0.54f	29.78 ± 0.59g
C18:2	10.96 ± 0.16a	51.13 ± 0.94b	23.56 ± 0.73c	55.44 ± 0.49d	31.18 ± 0.75e	14.62 ± 0.60f	32.63 ± 0.48g
C18:3	0.50 ± 0.06a	0.54 ± 0.06ab	6.20 ± 0.20c	0.23 ± 0.06d	0.34 ± 0.05d	2.14 ± 0.08e	0.24 ± 0.06e
C20:0	0a	0.23 ± 0.05b	0.46 ± 0.12cd	0.29 ± 0.08bd	0a	0.49 ± 0.09c	0.27 ± 0.08b
C20:1	0a	0a	1.20 ± 0.17b	0a	0a	0.45 ± 0.08c	0a
C22:0	0a	0a	0.34 ± 0.08b	0a	0a	0.18 ± 0.06c	0a
Others	0.50 ± 0.03a	0.20 ± 0.04b	0.49 ± 0.05acIK	0.13 ± 0.06b	0.33 ± 0.11c	0.48 ± 0.02a	0.22 ± 0.06b
SFA	46.21 ± 0.27	9.34 ± 0.05	8.01 ± 0.06	27.18 ± 0.16	31.83 ± 0.22	34.64 ± 0.22	36.96 ± 0.26
MUFA	41.66 ± 0.20	38.89 ± 0.62	60.44 ± 0.53	17.10 ± 0.27	36.05 ± 0.54	47.61 ± 0.35	30.04 ± 0.39
PUFA	11.34 ± 0.22	51.89 ± 0.45	31.41 ± 0.29	55.96 ± 0.22	31.52 ± 0.37	17.71 ± 0.26	33.14 ± 0.22
C18:2/C16:0	0.27 ± 0	8.76 ± 0.22	4.63 ± 0.05	2.37 ± 0.05	1.16 ± 0.04	0.48 ± 0.09	1.02 ± 0.02
Polyene index	0.25 ± 0.01	5.53 ± 0.17	3.72 ± 0.17	2.05 ± 0.04	0.99 ± 0.03	0.49 ± 0.03	0.89 ± 0.02
Iodine value (Wijs)	56.21 ± 0.61A	112.79 ± 0.85B	108.65 ± 1.04C	110.71 ± 0.70BC	85.53 ± 1.01D	71.68 ± 0.95E	82.60 ± 0.96F

Means within a row for the case of individual fatty acid marked with the same lowercase letters do not differ significantly at *P *<* *0.05. Means within a row for the case of iodine value marked with the same uppercase letters do not differ significantly at *P *<* *0.05. PO, palm olein; SFO, sunflower oil; CNO, canola oil; CSO, cottonseed oil; SFA, saturated fatty acids; MUFA, monounsaturated fatty acids; PUFA, polyunsaturated fatty acids.

Table 1 also shows the degree of polyunsaturation in parent and blended oils: linoleic to palmitic acids ratio (C18:2/C16:0) and the polyene index (ratio of polyunsaturated fatty acids to SFA). Reduction in those values was much expected when PO was added to SFO, CNO, and CSO. This trend was similar in the case of iodine value (IV), which correlates well with FAC. It is evidenced from Table 1 than lowering the C18:2/C16:0 and polyene index values serve for better oil stability. It is worth noting that these markers are normally used to measure the degree of polyunsaturation in oils and their tendency to undergo oxidative degradation.

It is also interesting to note a balance ratio between SFA, MUFA, and PUFA (∼1:1:1) was obtained in the case of PO + SFO and PO + CSO; the above ratio is in compliance with the recommendation by the American Heart Association (AHA) (Sundram et al. 2003). The remaining oil blend, however, did not meet the AHA recommendation when 30% CNO was added to 70% PO. The selection of this blending ratio was made in such a way that the concentration of linolenic acid does not exceed 2%. Oils that have more than 2% linolenic acid, for example in the case of CNO (Table 1), are not permitted for use in industrial frying (Berger 2005). Significant amount of linolenic acid not only imparts bitterness to the fried product but also releases unpleasant odor throughout the course of frying.

### Physico-chemical properties of oil during frying

#### Tocols

Tocols (tocopherols and/or tocotrienols) or commonly known as vitamin E are natural antioxidants that inherently present in oils. These vital constituents principally protect the oils by acting as radical scavengers to decelerate the propagation phases of oxidative degradation (Sánchez-Muniz et al. 2007). From Table 2, the initial tocols content in the four oils was insignificantly different even though the concentration of tocols in PO + CNO was slightly lower (497 mg kg^−1^). It is worth mentioning that most vegetable oils including SFO, CNO, and CSO only contain tocopherols whereby PO has a benefit of having tocopherols and tocotrienols. Indeed, PO is one of the richest sources of tocotrienols following palm kernel, coconut, and rice bran oils (de Marco et al. 2007). Therefore, blending the soft oils with PO, to some extent, provides opportunity to enrich the oils with tocotrienols.

**Table 2:** Changes of tocols, induction period, color, and *p*-anisidine value in oils during frying.

Oils	Parameters	Frying time (h)							
		0	8	16	24	32	40	48	56
PO	Tocols (mg kg^−1^)	587 ± 17aA	475 ± 23abD	393 ± 32cA	414 ± 28cAC	384 ± 17cA	401 ± 20cA	393 ± 30cA	379 ± 32cA
	Induction period (h)	21.4 ± 1.5aE	15.3 ± 1.0bcE	16.1 ± 0.7bE	14.6 ± 0.7cE	15.2 ± 0.6bcE	14.6 ± 0.7cE	15.2 ± 0.9bE	14.8 ± 1.1bE
	Color (R)	2.8 ± 0.2aI	8.3 ± 0.5bI	10.6 ± 0.7cI	11.7 ± 1.1cI	15.5 ± 0.6dI	18.1 ± 0.9eI	19.3 ± 0.5eI	21.2 ± 0.5fI
	*p*-anisidine (unit)	0.8 ± 0.1aM	29.4 ± 2.1bM	35.2 ± 2.7cM	36.6 ± 3.6cM	37.0 ± 2.3cM	33.2 ± 2.1cdM	31.4 ± 4.1cdM	35.6 ± 3.5cM
PO + SFO	Tocols (mg kg^−1^)	546 ± 15aB	497 ± 5bA	418 ± 11cA	402 ± 9cdA	374 ± 19dA	392 ± 18cA	383 ± 18cA	374 ± 15dA
	Induction period (h)	10.6 ± 0.2aF	9.2 ± 0.5abF	8.9 ± 0.2bF	8.9 ± 0.3bF	9.0 ± 0.5bF	8.6 ± 0.2bF	8.7 ± 0.3bF	8.6 ± 0.3bF
	Color (R)	1.4 ± 0.1aJ	4.1 ± 0.1bJ	5.1 ± 0.2cJ	6.4 ± 0.4dJ	6.6 ± 0.3dJ	7.2 ± 0.1eJ	8.3 ± 0.3fJ	9.5 ± 0.3gJ
	*p*-anisidine (unit)	2.3 ± 0.2aN	27.0 ± 1.3bM	38.5 ± 2.1cMN	33.8 ± 2.3dM	37.5 ± 2.5cdeM	40.7 ± 3.3cN	34.2 ± 2.0deM	39.0 ± 3.3cdeM
PO + CNO	Tocols (mg kg^−1^)	497 ± 29aC	397 ± 35bC	318 ± 26cdeB	333 ± 30dBD	276 ± 24eB	295 ± 47cdeB	267 ± 19eB	278 ± 17eB
	Induction period (h)	11.3 ± 0.9aF	9.1 ± 1.0bF	7.8 ± 0.8cFG	5.9 ± 0.5dG	6.2 ± 0.7dG	5.3 ± 0.5dG	5.7 ± 0.6dG	4.9 ± 0.9dG
	Color (R)	1.2 ± 0.1aJ	4.8 ± 0.3bK	8.6 ± 0.4cK	10.3 ± 0.7dK	11.9 ± 0.1efK	12.4 ± 0.4fK	12.0 ± 0.1fK	13.2 ± 0.4gK
	*p*-anisidine (unit)	4.0 ± 0.6aO	34.6 ± 2.3bN	41.2 ± 1.9cN	50.7 ± 3.5dN	59.0 ± 3.7eN	63.5 ± 5.3eO	58.1 ± 4.4eN	64.8 ± 2.9eN
PO + CSO	Tocols (mg kg^−1^)	523 ± 16aB	461 ± 22bD	484 ± 20bC	378 ± 17cCD	323 ± 19dC	305 ± 24dB	352 ± 31cdA	327 ± 18dA
	Induction period (h)	8.3 ± 0.5aG	7.0 ± 0.3bcG	7.5 ± 0.5abG	6.1 ± 0.5cG	4.0 ± 0.4dH	4.3 ± 0.5dG	3.7 ± 0.6dH	3.9 ± 0.5dG
	Color (R)	2.4 ± 0.3aK	5.2 ± 0.5bK	9.0 ± 0.4cK	11.0 ± 0.1dK	12.6 ± 0.4efL	12.1 ± 0.1eK	12.5 ± 0.5efK	12.8 ± 0.1fK
	*p*-anisidine (unit)	3.1 ± 0.4aO	28.6 ± 1.7bM	29.0 ± 3.0bM	46.2 ± 3.7cN	57.7 ± 2.4dP	47.1 ± 3.3cN	51.3 ± 4.1cdP	58.2 ± 5.3dP

Means within a row for the case of each parameter marked with the same lowercase letters do not differ significantly at *P *<* *0.05. Means within a column for the case of each parameter marked with the same uppercase letters do not differ significantly at *P *<* *0.05. PO, palm olein; SFO, sunflower oil; CNO, canola oil; CSO, cottonseed oil.

The change in tocols with the increase in frying times is also presented in Table 2. All oils experienced a gradual drop of tocols content during the first 32 h of frying, and achieve consistency thereafter. The rate of tocols loss was more pronounced in the case of PO + CNO (41%) whereby 37% in the case of PO + CSO, 35% in the case of PO, and 32% in the case of PO + SFO. Higher percentage of tocols retention in the latter oil is probably because of a significant amount of *α*-tocopherol present in the blend that exhibits greater antioxidant activity than other homologues. It is worth mentioning that SFO is the main source of *α*-tocopherol, which corresponds up to 90% of the total tocols (CODEX Alimentarius 2001).

#### Induction period

Induction period or so-called oxidative stability is an expression to describe the extent of oil stability by examines the time needed for oil to resist oxidation at elevated temperatures (Matthäus 2006). From Table 2, the induction period of fresh PO was the highest, while the oil blends displayed the values of below than 12 h. It is interesting to note that the induction period seems to improve with the increase in PO in the oil blends, that is, PO + CNO > PO + SFO > PO + CSO despite that the initial tocols content of the investigated oils was more or less comparable. This suggests that each tocol homologue, to some extent, exhibits different degree of antioxidant capacity. With regard to the fatty acids composition, it is also expected from Table 1 that equal amount of SFA in the oil blends (31.83% in the case of PO + SFO, 34.64% in the case of PO + CNO, and 36.96% in the case of PO + CSO) would result in similar induction periods, but this is not the case for the oil blends.

Results shown in Table 2 demonstrate a gradual decrease in the induction period of oils during the first stage of frying before hovering within a constant value throughout the remaining frying times, and this profile is more or less similar to those obtained in the tocols content. The induction period fell sharply by 31% from its initial value in the case of PO, 19% in the case of PO + SFO, 57% in the case of PO + CNO, and 53% in the case of PO + CSO, and achieved equilibrium states after frying for 24, 8, 40, and 32 h, respectively. Ahmad Tarmizi and Razali (2008) concluded that induction period has some form of correlation with tocols retention when comparing different grades of palm olein. This, however, may be only true for oils originating from the same source.

#### Color

Color is a subjective and one of the rapid parameter used to determine the quality of oils. In the light of starting oils, it is clearly evidenced in Table 2 that blending PO with SFO and CNO gave lighter oil color, which improved from 2.8 R to 1.4 R and 1.2 R, respectively. The PO + CSO, on the other hand, insignificantly changed the appearance of the oil (2.4 R). It is plausible that PO and CSO contain some trace of carotenoids, which in turn, contribute to the increase in redness value (Mohamed Sulieman et al. 2006).

Table 2 also shows the color transients of PO and the oil blends as a function of frying time. Rapid increase in oil color was obvious for the first 16 h of frying, followed by a steady increase in the redness value for the remaining frying times. As expected, PO gave the highest rate of color darkening (0.33 R h^−1^) whereas PO + SFO was the lowest (0.14 R h^−1^). The rate of color darkening was almost similar for the other two blends, that is, 0.19 R h^−1^ in the case of PO + CSO and 0.21 R h^−1^ in the case of PO + CNO.

Several publications have reported that PO darkening is due to the presence of trace phenolic compounds, many of which are natural antioxidants (Pantzaris 1997). It is also important to note that the quality of fried product is not affected albeit PO has relatively higher color value and darkens faster compared to other vegetable oils (Mohamed Sulieman et al. 2006; Koh et al. 2011). Darkening of oil during frying is generally associated with degradation processes involving the formation of hydroperoxides, conjugated dienoic acids, ketones, and hydroxides (Farhoosh et al. 2009). Other possible cause of color changes is the diffusion of browning pigments developed from potatoes during frying (Tsaknis et al. 2002).

#### *p*-Anisidine value

The *p*-anisidine value (AnV) is used to monitor the degree of oil oxidation during frying. This parameter determines the content of aldehydes when hydroperoxides, which are developed in the early stage of oxidation, decompose instantly when exposed to high frying temperatures (Pantzaris 1997). Indeed, AnV is said to be a reliable method to express the oxidative state of oil as aldehydes have higher resistance against heat as compared to hydroperoxides.

The initial AnV in the four oils was considerably low (no higher than four units) which further attests the quality of oils used in the study. In general, the AnV increased progressively just after 8 h of frying and subsequently demonstrated a moderate increase toward the end of frying time (Table 2). This could be due to the advanced stage of secondary oxidation in which constituents such as carbonyls, alcohols and fatty acids are subsequently react with oxygen to generate higher molecular weight compounds between fatty acids (carbon-to-carbon and/or oxygen-to-carbon bridges) (Tsaknis & Lalas, 2002). After 56 h of frying, the AnV was markedly high when frying was undertaken using blends of PO with CNO (64.8 unit) and CSO (58.2 unit), respectively. The other two oils, on the other hand, have similar AnV of which is lesser by more than one-third of the value obtained from the former oils.

#### Free fatty acid

**Figure 1:**
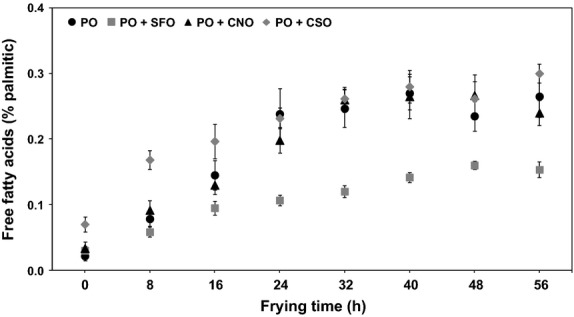
Changes of free fatty acid in oils during frying. One-way analysis of variance (ANOVA) was used to determine the significant different between oils for each frying time (*P *<* *0.05).

Free fatty acid (FFA) is basically developed when oils composition is hydrolytically altered as a result of reaction with moisture release from food, and partly due to decomposition of oils at frying temperatures (Al-Khusaibi and Niranjan 2012). Determination of FFA is relatively fast and reliable, and therefore this method is preferably used by many food processors to monitor the acidity of oils during frying (Ahmad Tarmizi and Ismail 2008). Fresh PO and the oil blends were of good quality when the FFA was only within a narrow range of 0.02% to 0.07% (Fig. 1); this value is still in compliance with the legislative FFA content of maximum 0.1% for refined edible oils (Basiron 2005).

Results shown in Figure 1 indicate a moderate increase in the FFA before reaching a steady state after 24 h for PO (0.24–0.27%), 32 h for PO + CNO (0.24–0.27%) and PO + CSO (0.26–0.30%), and 40 h for PO + SFO (0.14–0.16%). It is generally believed that oil inside the fryer—which is absorbed by the fried product and lost during frying—is compensated with fresh oil having lower FFA value, and thus yields in constant FFA content in frying oil (Ahmad Tarmizi and Ismail 2008). It is also noted from Figure 1 that the FFA level in the case of PO + SFO did not exceed 0.16% of which is considerably low compared to other oils. This finding is in agreement with the results obtained by Kristott (2000) after conducting a 20-h continuous frying operation using PO and SFO. The authors observed that the FFA of the former rose sharply by twofold from its initial value after 10 h of frying and reached consistency afterward; however, the latter only demonstrated a 14% increase in FFA although the content of the starting oil was almost five times higher than the former.

The threshold of FFA varies depending on the type of product being fried. For example, the FFA content of 0.5% for potato chips and instant noodles, and 1% for prefried French fries are normally regarded by food processors as bench marks for discarding used oils (Ismail 2005). Higher FFA contents of 2–2.5% are used as the limit for oils used for frying battered and breaded products. There are countries that even have their own legislative FFA limits for used oils: Germany (1%), Austria, and Japan (1.25%), the Netherlands (2.25%), and Belgium (2.5%) (Rossell 1997). By considering 0.5% as an upper limit, the FFA contents of all oils were well below the discard point, which further suggests that the frying operation can be extended for more than 56 h without the need to fully replace the oils inside the fryer.

#### Smoke point

Smoke point is the temperature of which the oil starts to produce a continuous wisp of bluish smoke when heating takes place. The presence of FFA is generally associated with the smoke point: increase in the FFA content lowers the smoke point value. This is based on the fact that the amount of smoke emanating from the oil is directly proportional to the concentration of low-molecular-weight constituents, for example, FFA, monoacylglycerols (MAG), diacylglycerols (DAG), and volatile compounds (Matthäus 2006).

**Figure 2:**
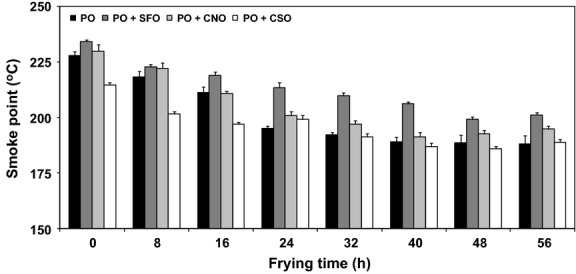
Profiles of smoke point in oils during frying. One-way analysis of variance (ANOVA) was used to determine the significant different between oils for each frying time (*P *<* *0.05).

Change in smoke point of oils across 56 h of frying operation is illustrated in Figure 2. The initial smoke point of PO + CSO was the lowest (215°C) while the remaining oils demonstrated higher smoke point values ranging from 228°C to 234°C. This is much expected when the initial FFA content is relatively high (0.07%; Fig. 1). It is recommended that the smoke point of the starting oil should be higher than 215°C, and with respect to this, all the above oils meet the specifications (Ahmad and Ismail 2007; Berger 2005). After 56 h of frying, the smoke point declined to 189°C for PO and PO + CNO, 195°C for PO + CNO, and 201°C for PO + SFO. Nevertheless, these values were still above the frying temperature (180°C) and in any case, 20°C higher than that of the minimum allowance adopted by some countries (Berger 2005).

#### Polar compounds

**Figure 3:**
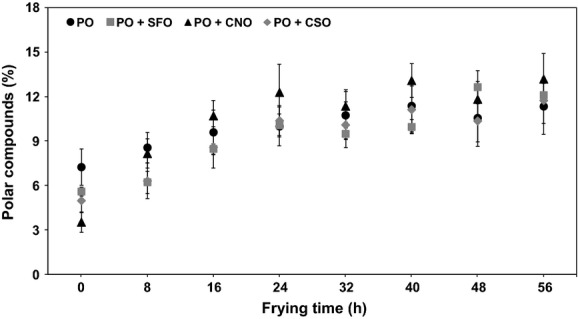
Formations of polar compounds in oils during frying. One-way analysis of variance (ANOVA) was used to determine the significant different between oils for each frying time (*P *<* *0.05).

Quantification of polar compounds is considered as the most objective method to examine the deteriorative effect in frying oils (Mohamed Sulieman et al. 2006). The polar compounds fractions—that is, polymerized and oxidized triacylglycerols (TAGs) and diacylglycerols (DAGs), and FFAs—are being developed during the oxidation and polymerization stages (Dobarganes et al. 2003). Figure 3 shows the trait of polar compounds with the increase in frying time. As expected, the polar compounds of fresh PO were the highest (7.3%); this can be explained on the basis of significant amount of DAGs presence in PO (Berger 2005). Blending PO with soft oils enables reduction in the level of polar compounds in the final oil: PO + SFO (5.6%), PO + CNO (3.5%), and PO + CSO (5.0%).

In light of frying stability, polar compounds rose sharply at the beginning of frying period and lingered constantly afterward within a narrow range of 10–11.4% for PO, 9.5–12.7% for PO + SFO, 11.8–13.2% for PO + CNO, and 10.1–11.8% for PO + CSO (Fig. 3). Interestingly, PO gave the lowest rate of formation of polar compounds despite the polar compounds of the starting oil being considerably high. Rossell (1997) emphasized that oils containing more than 25% are unfit for human consumption. This is supported by research which shows that the polar compounds fractions isolated from abused oils are extremely toxic to laboratory animals. For this reason, some countries dictate a regulation on the acceptance limit of polar compounds in used oils, for example, 25% for countries like Belgium, Chile, France, Italy, Spain, and South Africa, while 27% for countries like Austria and Germany (Berger 2005). In this study, all data showed in Figure 3 were well below the discard point.

#### Polymer compounds

Polymer compounds—which are a fraction of polar compounds—are developed through tertiary oxidation and thermal modification in oil structure when exposed to high temperatures: the latter is more prominent based on the fact that steam release from the product provides some form of protection to the frying oil by minimizing contact with oxygen (Gertz and Kochhar 2001). The formation of polymer compounds is responsible for the change in oil viscosity, tendency to foam during frying, and imparts bitterness to the fried product (Samah and Fyka 2002). Furthermore, viscosity would also cause a considerable amount of oil adhering onto the product surface and thus increase the oil content (Maskan and Bagci 2003).

**Figure 4:**
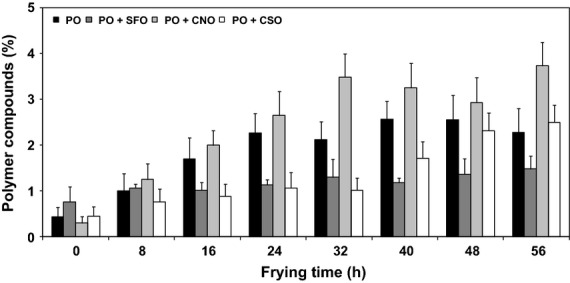
Developments of polymer compounds in oils during frying. One-way analysis of variance (ANOVA) was used to determine the significant different between oils for each frying time (*P *<* *0.05).

The development of polymer compounds across throughout the course of frying is shown in Figure 4. At the end of frying, the percentage of polymer compounds in PO + CNO was the greatest (3.7%), followed by PO + CSO (2.5%), and PO (2.3%). The formation of polymer compounds was minimal (1.5%) when PO + SFO were used as frying media. Nevertheless, all the values shown in Figure 4 are much lower than the maximum limit of polymer compounds, varying from 10% to 16%. Some European countries applied a stringent bench mark of 10% whereby countries like the Netherlands and South Africa have more lenient limit of polymer compounds (16%) in used oil (Ahmad and Ismail 2007).

## Conclusions

This study examined the physico-chemical changes in oil when palm olein was blended with sunflower, canola, and cottonseed oils in the form of binary mixtures. This is to improve oil stability before use under continuous frying conditions. Oil deterioration was relatively slow across frying times, and in most cases, the stability of the oil blends was equivalent to that of palm olein. Indeed, this finding provides useful information to food processors and consumers who are looking for stable oil for industrial frying without the need to fully replace their preferable local oils.
